# Using signal detection test to assess the correlation between daily sleep duration, mental workload, and attention sensitivity in nurses

**DOI:** 10.1186/s12912-024-02515-6

**Published:** 2024-11-21

**Authors:** Mohammad Hassan Kazemi-Galougahi, Ehsan Feizabadi

**Affiliations:** 1 Department of Social Medicine, Faculty of Medicine, AJA University of Medical Sciences, Tehran, Iran; 2 Faculty of Medicine, AJA University of Medical Sciences, Tehran, Iran

**Keywords:** Attention, Medical errors, Mental disorders, Nurses, Sleep, Workload, Signal detection test

## Abstract

**Background:**

Increased attention among healthcare workers, particularly nurses, is crucial for preventing medical errors and patient harm. This study uses an objective approach that overcomes the limitations of the subjective self-report measures employed in prior research on nurses’ attention.

**Methods:**

This cross-sectional study was conducted from January to March 2023 among 108 nurses at Besat Hospital in Tehran, Iran. The data collected included demographic information, the NASA Task Load Index (NASA-TLX) for mental workload assessment, and the CogLab signal detection test for attention sensitivity measurement.

**Results:**

The participants exhibited a median mental workload of 68.5 (IQR = 14.9) and a median attention sensitivity of 52.5 (IQR = 39.2). Daily sleep duration was positively correlated with attention sensitivity (*r* = 0.644, *p* < 0.001), whereas mental workload was negatively correlated with attention sensitivity (*r* = -0.655, *p* < 0.001). Men demonstrated greater attention sensitivity (*p* = 0.040), and women reported greater mental workload (*p* = 0.043).

**Conclusion:**

Reducing daily sleep duration and increasing mental workload can diminish nurses’ attention sensitivity. Prioritizing adequate sleep and implementing strategies to reduce mental workload are crucial for enhancing nurse performance and patient safety.

## Key points

**•** Insufficient sleep and excessive mental workload can significantly impair nurses’ attention.
**•** Female nurses may be particularly vulnerable to these factors.
**•** Implementing strategies to optimize sleep schedules and reduce mental workload is crucial for enhancing overall nurse performance and patient safety.

## Introduction

Attention is defined as “the set of evolved brain processes that leads to adaptive and effective behavioral selection.” It is considered a group of brain processes resulting in the selection of consistent and effective behaviors. Behavior can serve as a means to assess the involvement of brain processes in attention [1]. Nurses, particularly those working in intensive care units, often deal with critically ill patients and must constantly monitor their conditions. Even brief lapses in attention can result in medical errors that can seriously harm or even lead to the death of patients [2]. Nurses rely heavily on attention to execute tasks efficiently, make accurate clinical judgments, and ensure patient safety [3]. However, the inherent challenges of nursing work, characterized by long hours, irregular shifts, and high levels of stress, can significantly compromise nurses’ attentional capacity [4]. Decreased attention can also jeopardize healthcare workers’ health. According to the U.S. Centers for Disease Control (CDC), an estimated 600,000–800,000 needlestick injuries occur annually among U.S. healthcare workers, primarily owing to lapses in attention during routine tasks [5].

Sleep, a vital physiological process essential for cognitive restoration and function, is intimately linked to attention. Adequate sleep is crucial for maintaining alertness, enhancing mental performance, and supporting emotional regulation [6]. Conversely, sleep deprivation has been consistently associated with impaired attention, decreased cognitive flexibility, and increased errors [7]. Nurses often experience sleep disturbances due to demanding work schedules, leading to potential negative consequences for their attentional performance and patient care [8].

Mental workload, defined as the cognitive effort exerted to perform a task, is another critical factor influencing attention. A high mental workload can deplete cognitive resources, leading to attentional fatigue and reduced performance accuracy [9]. Mental workload is a frequently studied topic in psychology and behavioral research. The challenges and inconsistencies associated with defining and measuring mental workload have attracted significant scholarly attention. Excessive mental workload can impair work performance [10]. Nurses frequently face high mental workloads due to complex patient care, time pressures, and multitasking demands [11].

Nurses, as healthcare professionals operating in high-pressure environments, require a keen ability to focus on relevant activities while filtering out distractions. This cognitive skill, known as attention sensitivity, is paramount for effective patient care. Attentional lapses can lead to medical errors and compromise patient safety. Factors such as sleep duration and mental workload may influence nurses’ attention sensitivity. To better understand the relationships among these variables, a robust and reliable assessment tool is necessary. The Signal Detection Test offers a valuable methodology for measuring attentional sensitivity.

Almost all previous studies on nurses’ attention have relied on subjective self-report measures, such as questionnaires, which are susceptible to biases and may not accurately reflect actual attention levels. In contrast, our study employs an innovative, objective approach using the “signal detection test” to assess attention sensitivity. This method provides a more precise and reliable measure of attention, overcoming the limitations of previous studies.

While previous research has examined the individual effects of sleep deprivation and mental workload on attention [12], the interplay between these factors in the context of nursing has received increasing attention in recent years. Understanding the complex relationships among sleep, mental workload, and attention is crucial for developing effective strategies to optimize nurses’ cognitive function and enhance patient safety.

This study aimed to investigate the correlations among daily sleep duration, mental workload, and attention sensitivity among nurses.

## Methods

This cross-sectional analytical study was conducted among nurses employed at Besat Hospital in Tehran, Iran.

### Sample size and sampling design

The sample size was determined via the sample size guidelines for correlation analysis proposed by Bujang MA et al. [13]. Assuming an alpha level of 0.05, a power of 90%, a baseline correlation of 0, and an alternative correlation of 0.3, a required sample size of 112 participants was estimated. Ultimately, 108 nurses were recruited through stratified random sampling to a representative sample of all hospital nurses is included in the study. To conduct stratified random sampling, the nursing population was initially stratified into age and sex groups. The proportion of each group within the total population was subsequently determined. By multiplying these proportions by the estimated sample size of 112, the number of samples for each stratum was calculated. Finally, simple random sampling was conducted to select the specified number of individuals from each stratum.

### Inclusion and exclusion criteria

The inclusion criteria included a bachelor’s or master’s degree in nursing and at least two years of work experience. The exclusion criteria included a history of sleep disorders, sensory or motor impairments, attention disorders, and the use of sleeping pills or sedatives.

### Data collection

Data collection was conducted from January to March 2023. To conduct this study, two questionnaires were used: a demographic characteristics questionnaire and NASA’s Task Load Index (NASA-TLX). The participants completed these questionnaires after a brief explanation of the study. Additionally, a CogLab signal detection test was used to assess participants’ attention sensitivity following a detailed explanation of the procedure. Informed consent was obtained from all participants, and their data were maintained confidentially.

1. Demographic characteristics questionnaire

The demographic characteristics questionnaire captured age, sex, education level, and mean daily sleep duration over the previous week.

2. NASA’s Task Load Index (NASA-TLX).

To assess mental workload, the NASA Task Load Index (NASA-TLX) was administered. This questionnaire, the NASA Task Load Index (NASA-TLX), is a widely used tool for assessing individual workload perceptions. Developed by Hart and Steveland at NASA, it was refined through over 40 laboratory simulations over three years [14]. The NASA Task Load Index (NASA-TLX) assesses mental workload across six dimensions, namely, mental, physical, and temporal demands, along with performance, effort, and frustration. The NASA-TLX comprises 21 items. The participants rated six subscales, mental, physical, and temporal demands, performance, effort, and frustration, on a 0–100 scale. The participants were given detailed instructions for each of the six subscales prior to rating. Each subscale was rated on a 100-point scale, with increments of five. The participants subsequently engaged in 15 pairwise comparisons of the subscales, selecting the subscale with the greatest impact on workload. Each subscale selection in the pairwise comparisons assigned a weighted score. The product of each subscale’s weighted score and its raw score was divided by 15 to yield a mental workload score ranging from 0 to 100. This score represents the overall mental workload index. The NASA-TLX has demonstrated reliability and validity in numerous studies (Cronbach’s alpha = 0.72, test-retest reliability = 0.77, and correlations in convergent validity between 0.97 and 0.98) [14–16].

3. Signal detection test.

**Fig. 1:**
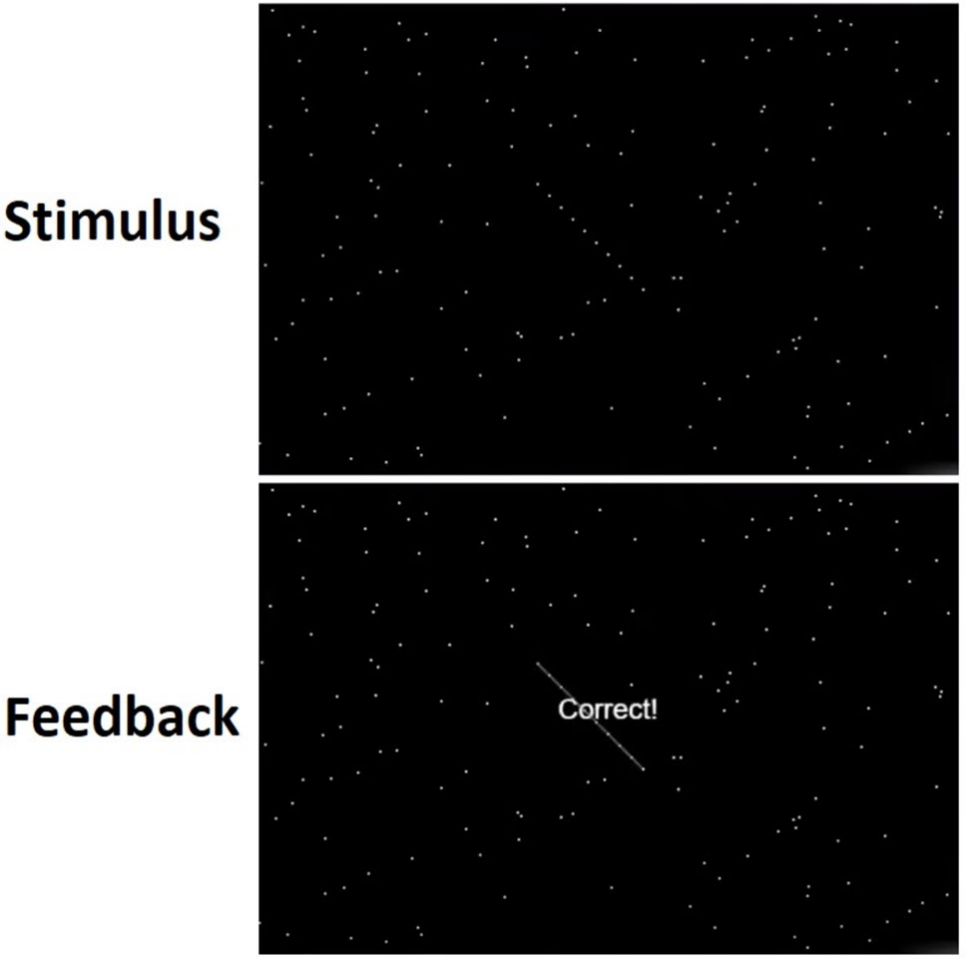
A view of how participants identified the target (a straight line of 10 dots) within the noise dots in the signal detection test

We used the CogLab signal detection test to assess attention sensitivity, and nurses participated in the test at the end of their shifts. The CogLab signal detection test, which is grounded in signal detection theory, was administered to participants [17]. This computer-based test comprised 60 trials, each displaying a varying number of “noise dots” on the screen. Each trial presented a random number of noise dots (144, 400, or 900) on the screen. Each noise dot quantity was presented in 20 trials. The participants determined the presence or absence of a target (a straight line of 10 dots) within the noise dots on each trial. (Fig. 1)

The target (a straight line of 10 dots) appeared in 50% of the trials, and 50% of the other trials had no target and presented only a random number of noise dots. Following each response, the participants received feedback on accuracy. A final d’ (d-prime) index quantified performance. The d-prime index measures a participant’s sensitivity to target detection. Typically, d-prime decreases as the number of noise dots increases [17].

### Statistical analysis

The data collected through the demographic characteristics questionnaire, NASA’s Task Load Index (NASA-TLX) and CogLab signal detection test were analysed statistically. Given the non-normal distribution of the quantitative data, the median and interquartile range (IQR) were used for descriptive analysis. Spearman’s rank correlation coefficient was used to examine correlations between daily sleep duration, mental workload, and attention sensitivity. The Mann‒Whitney U test was used to compare these variables between male and female nurses. Statistical significance was set at *p* ≤ 0.05.

## Results

One hundred and eight nurses participated in the study. The participants’ mean age was 28.9 years (standard deviation = 3.8). Table 1 presents the demographic characteristics of the study participants.

**Table 1:** Demographic characteristics of the participants (*n* = 108)

Variables	Groups	*N*	%
Gender	Male	48	55.6
	Female	60	44.4
Age group	< 30	78	77.2
	30–35	22	20.4
	≥ 36	8	7.4
Years of work experience	< 5	69	63.9
	6–9	36	33.3
	≥ 10	3	2.8
Educational level	Bachelor	99	91.7
	Master	9	8.3
Daily sleeping time (hour)	< 6	56	51.9
	6–7	29	26.9
	≥ 8	23	21.3

The combination of medium attention sensitivity and a high mental workload was most prevalent among the participants. Table 2 details the distributions of attention sensitivity and mental workload levels.

**Table 2:** Attention sensitivity and mental workload levels of the participants (*n* = 108)

Variables	Median (IQR)	Levels	*N*	%
Attention sensitivity	52.5 (39.2)	Low	27	25.0
		Medium	51	47.2
		High	30	27.8
Mental workload	68.5 (14.9)	Low	0	0
		Medium	48	44.4
		High	60	55.6

Among the mental workload subscales, mental demands had the highest median score (80.0 ± 15.0), whereas frustration had the lowest (40.0 ± 20.0). Table 3 presents the median and interquartile range (IQR) for each subscale.

**Table 3:** The medians and IQRs of the mental workload subscales of the participants (*n* = 108)

Mental workload subscales	Median	IQR
Mental Demands	80.0	15.0
Physical Demands	62.5	30.0
Temporal Demands	60.0	25.0
Own Performance	45.0	15.0
Effort	60.0	20.0
Frustration	40.0	20.0

**Fig. 2:**
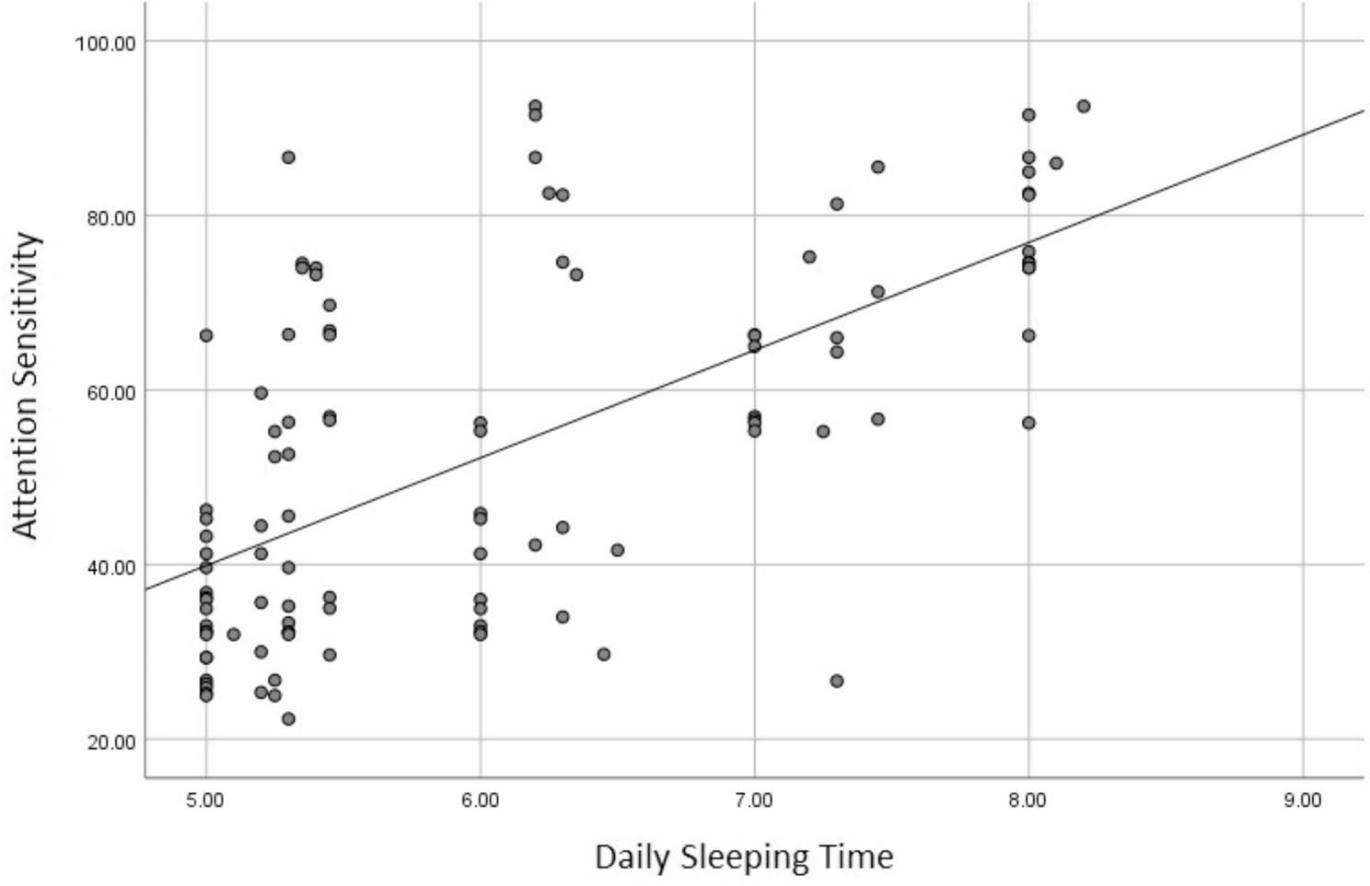
The correlation between daily sleeping time and attention sensitivity

**Fig. 3:**
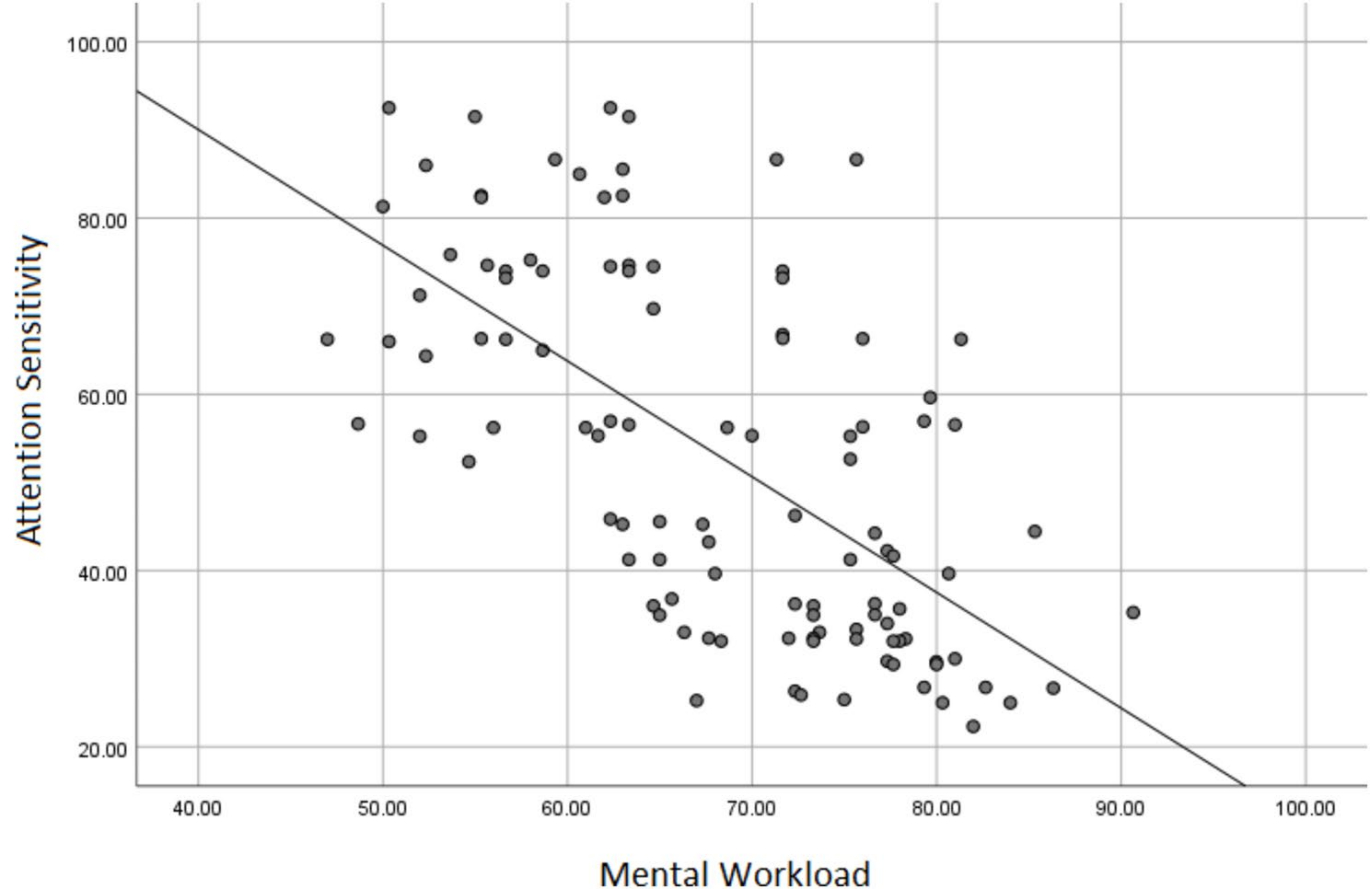
The correlation between mental workload and attention sensitivity

Daily sleep duration was positively correlated with attention sensitivity (*p* < 0.001), whereas mental workload was negatively correlated with attention sensitivity (*p* < 0.001). Table 4; Figs. 2 and 3 illustrate these correlations.

**Table 4:** The correlation between daily sleeping time and mental workload with attention sensitivity

Variables	Spearman’s Rank correlation coefficient	*P*-value
Daily sleeping time	0.644	< 0.001
Mental workload	-0.655	< 0.001

All six mental workload subscales (mental demands, physical demands, temporal demands, own performance, effort, and frustration) correlated negatively with attention sensitivity (*p* < 0.001). Effort exhibited the strongest negative correlation (*r* = -0.509), whereas temporal demands had the weakest (*r* = -0.344). Table 5 presents these correlations.

**Table 5:** Correlations between mental workload subscales and attention sensitivity

Mental workload subscales	Spearman’s Rank correlation coefficient	*P*-value
Mental Demands	-0.450	< 0.001
Physical Demands	-0.427	< 0.001
Temporal Demands	-0.344	< 0.001
Own Performance	-0.445	< 0.001
Effort	-0.509	< 0.001
Frustration	-0.448	< 0.001

Men exhibited significantly greater attention sensitivity (*p* = 0.040) than women did, whereas women reported significantly greater mental workload (*p* = 0.043). No significant difference in daily sleep duration was observed between the sexes (*p* = 0.630). Table 6 presents the medians and interquartile ranges of attention sensitivity, mental workload, and daily sleep duration by sex.

**Table 6:** The medians and IQRs of attention sensitivity, mental workload and daily sleeping time in participants by sex

Variables	Gender	Median	IQR	*P*-value
Attention sensitivity	Male	65.5	40.1	0.040
	Female	44.3	26.5	
Mental workload	Male	64.8	15.2	0.043
	Female	72.0	14.4	
Daily sleeping time	Male	6.0	2.2	0.630
	Female	5.4	1.2	

## Discussion

The findings of this study revealed a direct correlation between attention sensitivity and the quantity of daily sleep, as well as an indirect correlation with mental workload. Given that the CogLab signal detection test was administered at the end of shifts and that a quarter of nurses displayed low attention, healthcare administrators should implement interventions to increase nurse attention and concentration, thereby mitigating human factor-related medical errors.

All the participants reported moderate to high mental workload. Mental demands constituted the most influential subscale within the NASA-TLX. Given the significant negative correlation between mental workload and attention sensitivity, reducing mental workload can indirectly increase nurses’ attention. This finding aligns with previous research by Sungchul Mun [18].

Daily sleep duration correlated positively with attention sensitivity, emphasizing the importance of adequate sleep for nurses to optimize attention and reduce medical errors. These findings align with previous research by Soomi Lee [19] and Marco Di Muzio [20]. Some nurses are currently employed at two or more medical centers simultaneously, often driven by financial needs, which more common in recent years because of high inflation. Such demanding work schedules can lead to consecutive work shifts for these nurses, resulting in reduced daily sleep duration. Our findings indicate a direct correlation between daily sleep hours and attention sensitivity and that insufficient sleep can impair attention, potentially contributing to an increase in medical errors in these nurses.

Another factor that significantly impacts nurses’ sleep quality is the rotation of their work shifts. Numerous studies have demonstrated that nurses working rotating shifts experience poorer sleep quality than do those working fixed shifts [21–24]. Given the direct correlation between sleep quality and attention sensitivity, it is advisable to adjust work shift schedules in medical centers to minimize rotation shifts.

Interestingly, men presented greater attention sensitivity despite having sleep durations comparable to those of women. Conversely, women reported significantly greater mental workload. These findings underscore the need for targeted interventions to address the unique challenges faced by female nurses, such as workload reduction and potential sleep optimization strategies. Numerous studies have confirmed that women have greater mental workloads than men do [25, 26].

Ethical concerns surrounding the mental workload and sleep duration of nurses are paramount. Frequent or rotating shifts can lead to reduced sleep, which not only causes physical and mental health problems in nurses but also impairs their concentration and impacts patient safety. Healthcare organizations have a moral obligation to provide a safe and supportive work environment for their employees. This includes ensuring that nurses have adequate opportunities for rest and recovery. In addition, medical centers should implement strategies to manage nurses’ mental workloads effectively, such as optimizing staffing levels and distributing tasks equitably.

### Limitations and recommendations

We initially planned to conduct a linear regression model for multivariate analysis. However, due to the non-normal distribution of the dependent variable (attention sensitivity), a fundamental assumption of linear regression, we were compelled to conduct univariate analysis using non-parametric tests. Additionally, the inconsistent nature of the studied nurses’ work shifts, coupled with overlapping work schedules, precluded the inclusion of work shifts as an independent variable.

Future research should investigate the relationship between sleep, workload, attention, and medical error incidence among nurses and other healthcare professionals.

## Conclusion

The findings of this study indicate a significant negative correlation between mental workload and attention sensitivity among nurses. Additionally, adequate sleep duration was positively associated with heightened attention sensitivity. These results underscore the importance of optimizing sleep schedules and implementing strategies to reduce mental workload among nursing staff to increase overall attention and performance.

## Data Availability

The datasets used and analyzed during this study are available from the corresponding author on reasonable request.

## Declarations

### Relevance to clinical practice

To increase nurses’ attention and reduce medical errors, healthcare organizations should prioritize adequate sleep schedules and implement strategies to mitigate mental workload.
